# Evidence for characteristic vascular patterns in solid tumours: quantitative studies using corrosion casts

**DOI:** 10.1038/sj.bjc.6690416

**Published:** 1999-05

**Authors:** M A Konerding, W Malkusch, B Klapthor, C van Ackern, E Fait, S A Hill, C Parkins, D J Chaplin, M Presta, J Denekamp

**Affiliations:** Department of Anatomy, Johannes Gutenberg-University Mainz, Becherweg 13, Mainz, D-55099, Germany; Carl Zeiss Vision GmbH, Oskar-von-Miller-Str. 1, Eching, D-85386, Germany; Gray Laboratory Cancer Research Trust, P.O. Box 100, Northwood, Middlesex, HA6 2JR, UK; Department of Biochemical Sciences and Biotechnology, School of Medicine, Via Valsabbina 19, Brescia, I-25123, Italy; Oncology Department, Umeå University, Umeå, S-901-85, Sweden

**Keywords:** tumour, rodents, xenografts, vascular pattern, microvascular corrosion casting

## Abstract

The vascular architecture of four different tumour cell lines (CaX, CaNT, SaS, HEC-1B) transplanted subcutaneously in mice was examined by means of microvascular corrosion casting in order to determine whether there is a characteristic vascular pattern for different tumour types and whether it differs significantly from two normal tissues, muscle and gut. Three-dimensional reconstructed scanning electron microscope images were used for quantitative measurements. Vessel diameters, intervessel and interbranch distances showed large differences between tumour types, whereas the branching angles were similar. In all tumours, the variability of the vessel diameters was significantly higher than in normal tissue. The quantitative data provide strong evidence for a characteristic vascular network determined by the tumour cells themselves. © 1999 Cancer Research Campaign


*British Journal of Cancer* (1999) **80**(5/6), 724–732
© 1999 Cancer Research Campaign
Article no. bjoc.1998.0416

### Evidence for characteristic vascular patterns in solid tumours: quantitative studies using corrosion casts

**MA Konerding[1], W Malkusch[2], B Klapthor[1], C van Ackern[1], E Fait[1], SA Hill[3], C Parkins[3], DJ Chaplin[3], M Presta[4] and J Denekamp[5]**

[1]Department of Anatomy, Johannes Gutenberg-University Mainz, Becherweg 13, D-55099 Mainz, Germany; [2]Carl Zeiss Vision GmbH, Oskar-von-Miller-Str. 1, D-85386 Eching, Germany; [3]Gray Laboratory Cancer Research Trust, P.O. Box 100, Mount Vernon Hospital, Northwood, Middlesex HA6 2JR, UK; [4]Department of Biochemical Sciences and Biotechnology, School of Medicine, University of Brescia, Via Valsabbina 19, I-25123 Brescia, Italy; [5]Oncology Department, Umeå University, S-901-85 Umeå, Sweden

**Summary** The vascular architecture of four different tumour cell lines (CaX, CaNT, SaS, HEC-1B) transplanted subcutaneously in mice was examined by means of microvascular corrosion casting in order to determine whether there is a characteristic vascular pattern for different tumour types and whether it differs significantly from two normal tissues, muscle and gut. Three-dimensional reconstructed scanning electron microscope images were used for quantitative measurements. Vessel diameters, intervessel and interbranch distances showed large differences between tumour types, whereas the branching angles were similar. In all tumours, the variability of the vessel diameters was significantly higher than in normal tissue. The quantitative data provide strong evidence for a characteristic vascular network determined by the tumour cells themselves.

**Keywords:** tumour; rodents; xenografts; vascular pattern; microvascular corrosion casting

The vascular system plays a role of key importance during tumour growth and metastasis formation. In addition, the effectiveness of almost all therapeutic modalities, including drug therapy and radiotherapy, is influenced by the micro-architecture and the gradients of essential nutrients around each vessel. This underlines the importance of the vascular architecture, its origin and effectiveness as a nutrient delivery system. The knowledge that tumour vasculature is abnormal has led to concepts such as angiogenic attack and vascular targeting (Folkman, 1976; Denekamp, 1984; Bicknell, 1994; Folkman and D'Amore, 1996). This, in turn, has led to many more studies of tumour vasculature and primary as well as secondary angiogenesis. However, even though our knowledge of the mechanisms underlying angiogenesis has increased dramatically in the past 25 years, few quantitative data are available on the vascular network architecture and pattern formation in tumours.

The fact that the tumour vascularity differs in many aspects from the vasculature of normal organs and tissues was already recognized in the last century (Virchow, 1863; Thiersch, 1865). Thomlinson and Gray (1955) indicated the importance of intervessel spacing because of the threefold increase in radioresistance that accompanies reduction of $pO_2$ concentrations below critical level. The important question as to whether the vascular architecture of an individual tumour is tumour type-specific has been controversial (Warren, 1979; Vaupel and Gabbert, 1986.) This is due, at least in part, to the methodologies used. Most reports confine themselves to qualitative observations and comparisons of gross vascular patterns in host and tumour, or to blood vessel density, length and diameter measurements, which in turn vary with the staining and counting techniques (Davidson et al, 1994; Endrich and Vaupel, 1998). Recently, morphometric analyses have been introduced in which the features of tumour cells (proliferation rate, oxygenation, angiogenic growth factor production) have been mapped by sequential staining of the same section, allowing the influence of the vascular assay on clinically relevant aspects of cell populations to be mapped. Vascular and metabolic profiles (VAMP) have illustrated marked differences between different types of tumour of the same general histology, for brain tumours and those from the head and neck region.

Less et al (1991) introduced the first suitable approach for determining branching patterns and vessel dimensions in corrosion casts of mammary carcinomas. They established a quantitative classification scheme which takes account of the unique features of tumour microvascular network topology. However, it should be recognized that measurements were made on planar two-dimensional (2D) projected images of three-dimensional (3D) specimens. All attempts to determine distances in corrosion casts geometrically after 2D projection inevitably include a considerable error. Measurements of vascular parameters such as intercapillary distance and vessel segment length are only possible considering the spatial information. One method to receive correct dimensions is the 3D reconstruction of stereo pairs with defined tilt angle.

Against this background we used a recently introduced method for 3D measurements in microvascular corrosion casts for our studies (Malkusch et al, 1995). The aim in the present study was to compare qualitatively and quantitatively the microvascular patterns of four different experimental tumours and to compare them with two normal tissues.

*Received 13 July 1998*
*Revised 27 November 1998*
*Accepted 10 December 1998*

*Correspondence to:* MA Konerding

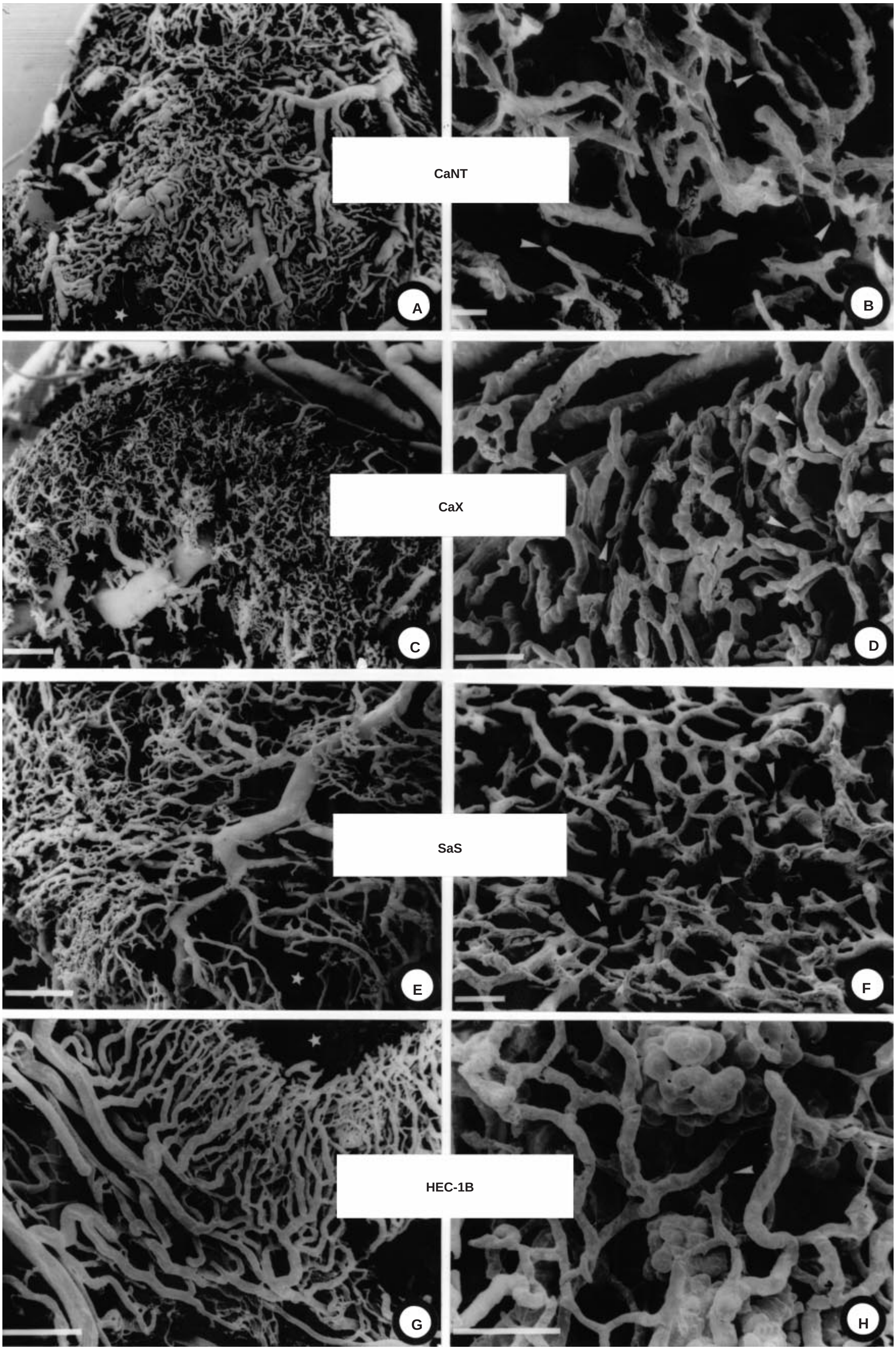

**Figure 1** Scanning electron micrographs of vascular corrosion casts from four different types of tumours. Left hand panels are low power magnifications (bars = 500 μm) illustrating tumour type specific differences of both the surrounding vascular envelope and the peripheral tumour vessels. All tumours lack a hierarchy of the vasculature. Note that **C** is a cross-section. The centrally located vessels in the right-hand panels (bars = 100 μm) also show differences in the pattern formation. * = avascular area; e = evasate; arrowheads = blind ends

*© Cancer Research Campaign 1999* 

### MATERIALS AND METHODS

### Tumour cell lines and animals

Four different tumours were used: two murine carcinomas (CaX, CaNT), a slow growing murine sarcoma (SaS) grown in the strains of origin, and a human endometrial adenocarcinoma grown in nude mice (HEC-1B). Crude suspensions of CaNT and CaX tumour cells, or 1 $mm^3$ pieces of solid SaS tumour, were transplanted subcutaneously into the back of five to seven 6- to 8-week-old CBA/Gy f TO mice (inbred for several decades, and syngeneic since the tumours had arisen spontaneously in this substrain over 5 years earlier) at the Gray Laboratories of the Cancer Research Campaign (Northwood, UK). Animals were bred and maintained in specific pathogen-free, category four conditions in a barrier-controlled animal house facility. The HEC-1B cells were transplanted behind the right foreleg of female NCr-*nu*/*nu* mice and grown as xenografts (obtained from the Animal Production Colony of the NCI-Frederick Cancer Research and Development Center, Frederick, MD, USA). Tumour inoculations resulted in a high incidence of tumour take and growth. The tumour sizes were measured three times a week with calipers. At 14–82 days, when these casting experiments were performed, the tumour volumes ranged between 0.15 and 0.9 ml (Table 1). The SaS and CaX were slow-growing and the CaNT and HEC-1B tumours were fast-growing.

In addition, seven extra mice, without implanted tumours, were used to obtain specimens of the vasculature of the subserosa of the gut and a standard striated rectus femoris muscle.

The procedures involving animals and their care were conducted in conformity with the institutional guidelines that are in compliance with national and international laws and policies (UK Animals Act: Scientific Procedures, 1986; EEC council directive 86/609, OJ L 358, 1, Dec. 12, 1987; NIH guide for the care and use of laboratory animals, NIH publication no. 85–23, 1985).

### Corrosion casting

All animals were thoracotomized under deep pentobarbital anaesthesia. The left ventricle was cannulated and the ascending aorta was entered with an olive-tipped cannula. The entire vasculature of the animal was thoroughly rinsed by perfusing with lukewarm saline (20–30 ml) and then fixed by perfusing with 2.5% buffered glutaraldehyde (20–25 ml, 860 mosmol, pH 7.40). Finally, up to 40 ml Mercox CL-2B (Vilene Med. Co., Tokyo, Japan) diluted with 20% methylmethacrylate monomers (Merck, Darmstadt, Germany) was gently perfused as a casting medium (Konerding et al, 1989). After complete polymerization in a lukewarm water bath the tumours were excised. In mice without tumours, parts of the large intestine and of the rectus femoris muscle were sampled.

The specimens were macerated in 5% potassium hydroxide, rinsed, dried and mounted on stubs for scanning electron microscopy. The microvascular corrosion casts were viewed after coating with gold in argon atmosphere with a Cambridge Stereoscan 180 scanning electron microscope (Cambridge, UK). From all specimens, peripheral and central areas were recorded as stereo images using a tilt angle of 6°.

### Morphometry and statistical analysis

Pairs of stereo images were used after digitization, image enhancement and 3D reconstruction with an image analysis program (Kontron KS 300, Kontron, Eching, Germany) to calculate parameters that describe the microvascular network architecture such as the intervessel and interbranch distances as well as the branching angles. For details of the reconstruction and calculation, see Malkusch et al (1995). For each tumour type, 351 to 1012 distances were calculated. In addition the vascular diameters and the variation of the diameter in individual vessel segments were assessed in 119 to 499 microvessels of each tumour type. The variation of the vascular diameter was assessed by measuring the diameter of a vessel segment, i.e. within the individual vessel's length between two branches, at four (CaX, SaS, CaNT) or five (HEC-1B) distinct points and expressed as the percentage deviation from the mean of these four or five measurements.

Statistical analyses and graphic displays were performed using SigmaStat and SigmaPlot (Jandel Scientific Software, Erkrath, Germany). All groups were tested for the form of their frequency distribution before analysis. The differences in the frequency distributions were tested for significance using the $\chi^2$ test, whereas the testing for significant differences using the mean and standard

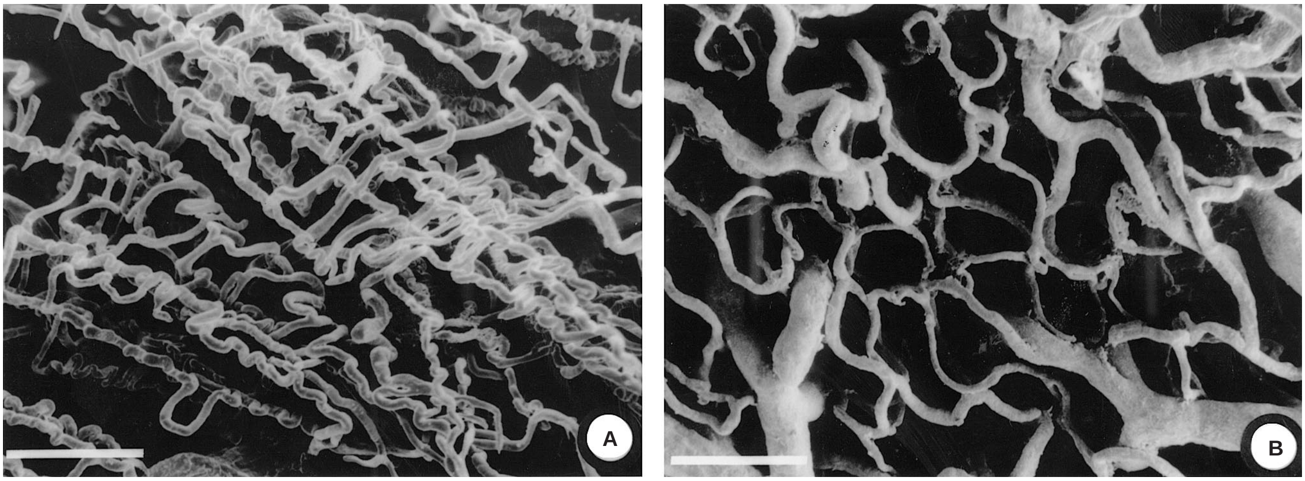

**Figure 2** Scanning electron micrographs of corrosion cast specimens of the vascular network of the skeletal muscle **(A)** with characteristic vessel courses and of subserosal capillaries of the gut **(B)** draining into venules. Bars = 100 μm

 © Cancer Research Campaign 1999

deviation of the distribution was performed with the Mann–Whitney rank sum test and analysis of variance (ANOVA). In all cases, the sensitivity was assessed by means of a power calculation, which revealed *d* values higher than 0.8 at the 5% level of confidence.

### RESULTS

### Vascular architecture

The vasculature was so characteristic for the individual tumours that the tumours belonging to any group could be identified with high certainty even at low power magnifications (Figure 1 A,C,E,G). All tumours were surrounded by vascular envelopes with high vascular densities formed by condensation of pre-existing host vessels. The extent of tumour-induced vessel dilations (Figure 1 A,E), compressions and tortuous courses depended on the tumour type. CaNT tumours, for example, regularly induced a peripheral plexus of dilated and tortuous courses (Figure 1A), whereas the periphery of SaS sarcomas was characterized by a more loosely woven network of draining veins (Figure 1E). Most of the peripheral vessels originate from veins (Figure 1 A,E,G). Arteries and arterioles, recognizable in corrosion casts because of their typically elongated endothelial cell nuclei imprints, were

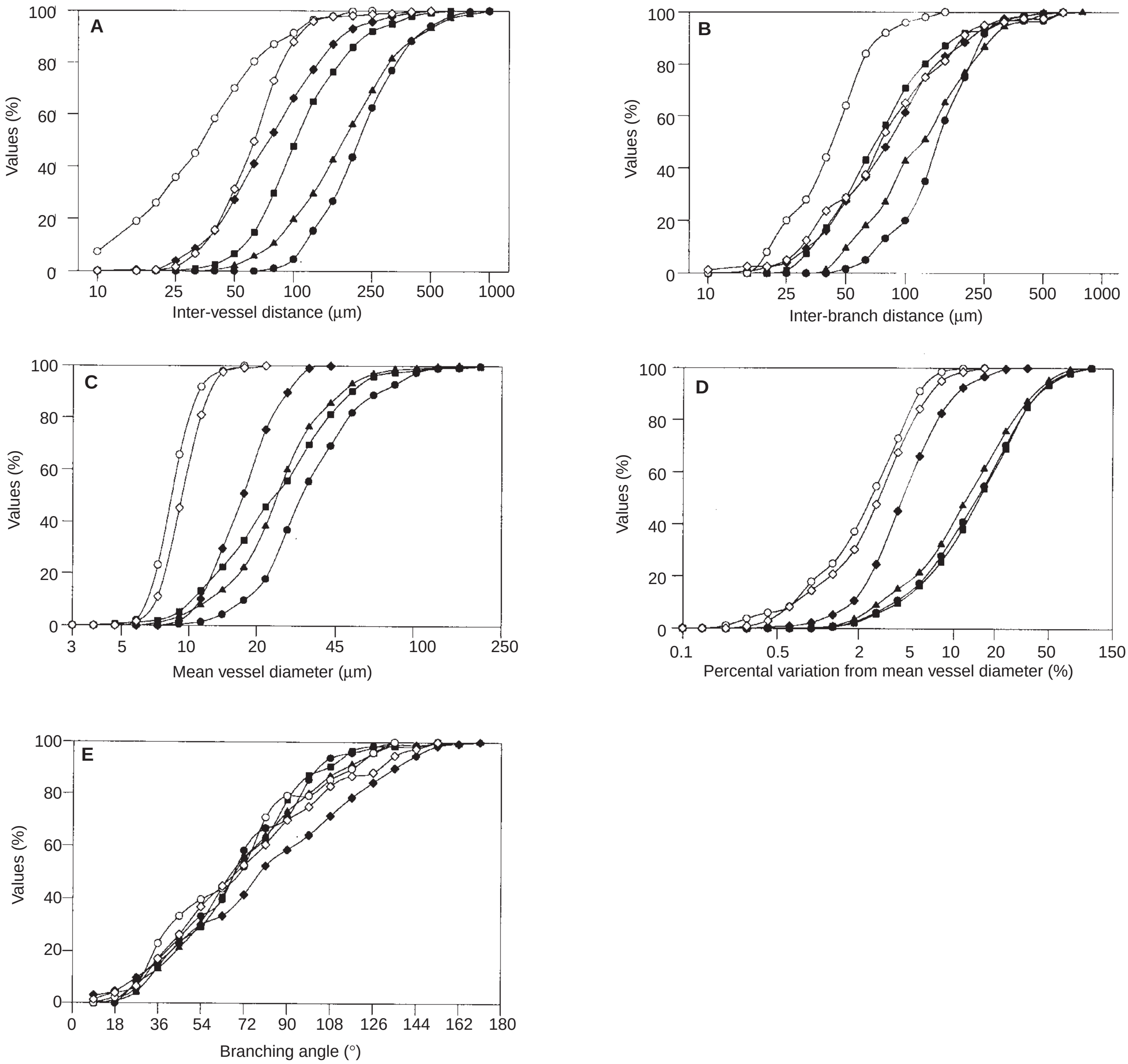

**Figure 3** Cumulative frequency distribution plots of different aspects of the vascular architecture in different tumours and normal tissues. ● = CaX, ▲ = CaNT, ■ = SaS, ◆ = HEC-1B tumours, ○ = musculature, ◇ = subserosal gut vessels. For median values, dispersion factors and numbers of measurements, see Table 2; for significance figures, see Table 3. **(A)** Inter-vessel distances: The distances between adjacent vessels show both a significantly greater spread of values and higher median values for all four tumours relative to the normal tissues. **(B)** Inter-branch distances: The distance between subsequent vessel branches is much shorter and less variable in muscle than in the four tumours and the gut serosa. **(C)** Vessel diameters: The mean vessel diameter is 2–4 times higher in tumours than in normal tissues and has a greater spread of values. **(D)** Percental variation of vessel diameters: Vessel diameters within individual vessel segments, i.e. between two consecutive branchings, have a two- to tenfold greater variation in tumours relative to normal tissues. Compare with Figure 4. **(E)** Branching angles: Regardless of the origin of the vascular networks the distribution of the branching angles shows wide similarities

*© Cancer Research Campaign 1999*

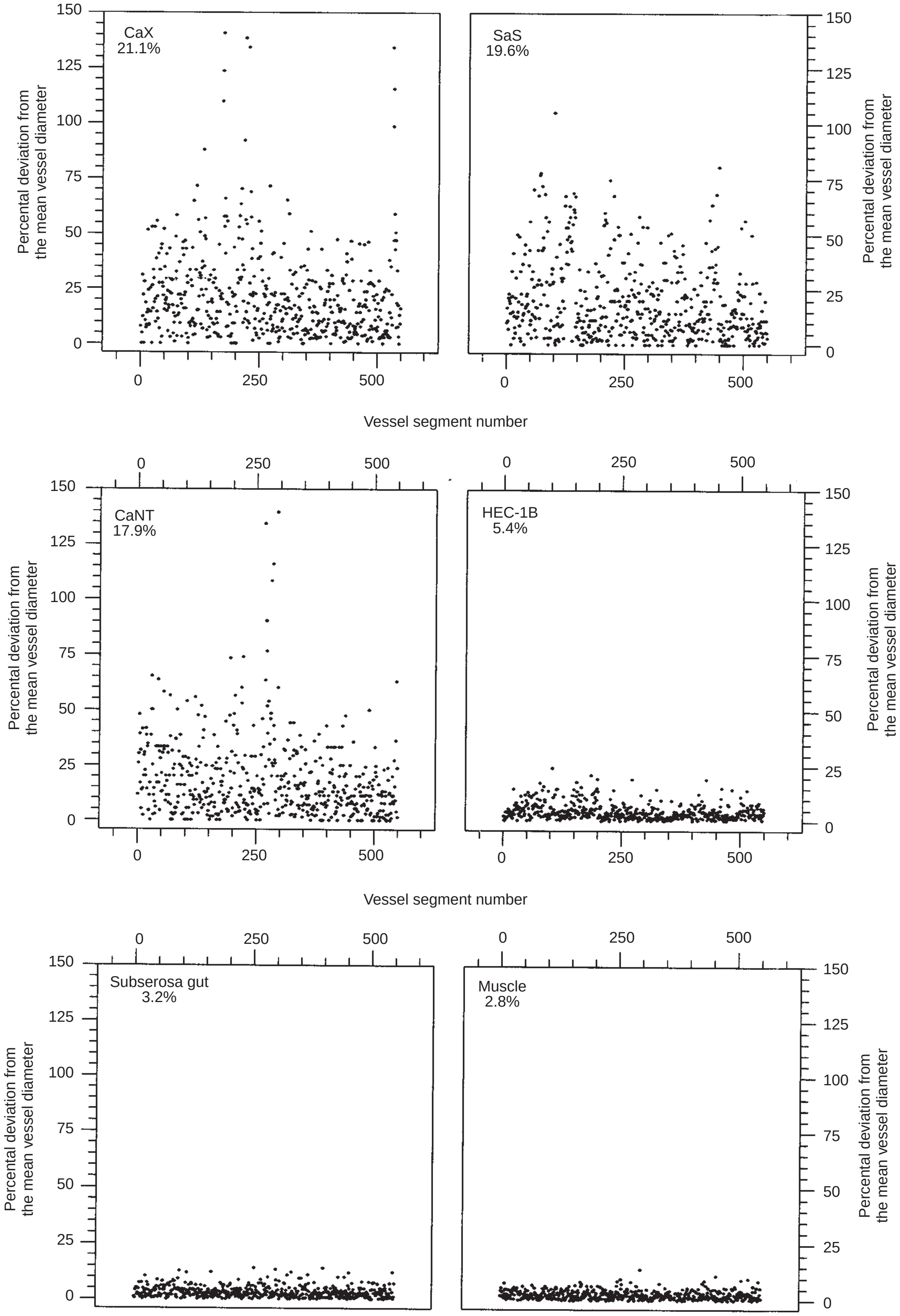

**Figure 4** Spectrum of the variation of vessel diameter within individual vessel segments in tumours and normal tissues. The per cent deviations from the mean diameter of four or five distinct points were calculated for each vessel segment. Between 99 and 499 vessel segments were measured. Because of better readability, the scattergrams depict maximally 550 values. The small fraction of values higher than 150% was not plotted. Note the much greater inhomogeneity in tumours than in normal tissues. The mean vessel diameter variation is shown in the upper lefts, and illustrates the much greater inhomogeneity in tumours than in normal tissues

*© Cancer Research Campaign 1999*

**Table 1** Tumour cells, number of injected cells, days after transplantation, and tumour volumes

| | No. of injected cells (× $10^6$) | Days pt | Tumour volume (ml; mean ± s.d.) | No. of examined casts[a]/ No. of animals |
|---|---|---|---|---|
| CaX | 1 | 65 | 0.91 ± 0.37 | 5/6 |
| SaS | 1 | 82 | 0.38 ± 0.12 | 4/6 |
| CaNT | 1 | 21 | 0.18 ± 0.03 | 5/7 |
| HEC-1B | 10 | 14 | 0.15 ± 0.06 | 4/5 |

[a]Only tumours that yielded excellent fillings of the vasculature were utilized for morphometric analysis.

seen to a far lesser extent. Even the tumour vascular envelope and the tumour periphery displayed heterogeneous vessel distributions (Figure 1 A,E,G). Only CaX tumours developed a homogeneous network in the periphery (Figure 1C). In two HEC-1B tumours the peripheral vascular envelope was incomplete (Figure 1G) and the newly formed peripheral vessels did not cover all aspects of the tumour at 2 weeks, the time of perfusion.

The network of the deeper microvasculature of each tumour is mainly made up of sinusoidal vessels with varying diameters, numerous blind ends, and lacking hierarchy (Figure 1 B,D,F). These networks clearly differ in many features from the vasculature in normal tissues (Figure 2 A,B). The chaotic pattern of CaNT tumours (Figure 1B) is characterized by lacunar vessels with large intervessel distances. CaX tumours frequently formed basket-like patterns (Figure 1D), whereas the pattern of SaS tumours was determined by dichotomous branching of the sinusoids (Figure 1F). In marked contrast to the syngeneic rodent tumours, the arrangement of the microvessels of the HEC-1B xenografts looked nearly normal, as evidenced by the more regular branching pattern and the lower frequency of diameter changes. However, numerous extravasates were observed in this tumour type, indicative of leaky sprouts and/or inadequately formed vessels.

In all tumour types heterogeneous vascular densities with large avascular areas were identified but showed no obvious correlation to differences in size or growth rate.

**Table 2** Median values (M), dispersion factors (DF) and numbers (N) of measured vascular parameters

| | | CaX | SAS | CaNT | HEC-1B | Gut | Muscle |
|---|---|---|---|---|---|---|---|
| Intervessel | M | 112.4 | 180.7 | 213.6 | 76.1 | 63.4 | 35.2 |
| distance | DF | 1.77 | 1.91 | 1.64 | 1.91 | 1.58 | 2.16 |
| (µm) | N | 535 | 777 | 291 | 234 | 188 | 174 |
| Interbranch | M | 69.7 | 125.0 | 147.9 | 82.3 | 76.3 | 42.6 |
| distance | DF | 1.92 | 1.93 | 1.65 | 2.02 | 1.88 | 1.84 |
| (µm) | N | 202 | 235 | 60 | 277 | 135 | 182 |
| Vessel | M | 24.4 | 24.3 | 32.0 | 17.5 | 8.4 | 9.4 |
| diameter | DF | 1.88 | 1.68 | 1.69 | 1.39 | 1.22 | 1.25 |
| (µm) | N | 1440 | 1996 | 1756 | 595 | 630 | 495 |
| Variation of | M | 15.4 | 11.1 | 14.3 | 4.2 | 2.7 | 2.4 |
| vessel diameter | DF | 2.56 | 2.60 | 2.52 | 2.01 | 2.24 | 2.26 |
| (%) | N | 1440 | 1440 | 1440 | 550 | 630 | 495 |
| Branching | M | 68.3 | 67.1 | 68.3 | 79.5 | 70.5 | 68.7 |
| angle | DF | 1.56 | 1.66 | 1.56 | 1.96 | 1.42 | 1.34 |
| (°) | N | 116 | 160 | 48 | 328 | 144 | 122 |

**Table 3** Significance figures of the measured parameters

| | Intervessel distance | Interbranch distance | Vessel diameter | Variation of vessel diameter | Branching angle |
|---|---|---|---|---|---|
| CaNT vs CaX | *** | *** | *** | NS | NS |
| CaNT vs SaS | *** | * | *** | NS | NS |
| CaNT vs HEC-1B | *** | *** | *** | *** | NS |
| CaNT vs muscle | *** | *** | *** | *** | NS |
| CaNT vs gut | *** | *** | *** | *** | NS |
| CaX vs SaS | *** | *** | NS | NS | NS |
| CaX vs HEC-1B | *** | NS | *** | *** | NS |
| CaX vs muscle | *** | *** | *** | *** | NS |
| CaX vs gut | *** | NS | *** | *** | NS |
| SaS vs HEC-1B | *** | *** | *** | *** | NS |
| SaS vs muscle | *** | *** | *** | *** | NS |
| SaS vs gut | *** | *** | *** | *** | NS |
| HEC-1B vs muscle | *** | *** | *** | ** | NS |
| HEC-1B vs gut | * | NS | *** | ** | NS |
| Muscle vs gut | *** | *** | ** | NS | NS |

NS = not significant; *$P \leq 0.05$; **$P \leq 0.005$, ***$P \leq 0.0001$.

*© Cancer Research Campaign 1999*

### Morphometric data of the microvascular architecture

The above mentioned qualitative differences in the macroscopic and microscopic arrangement of the microvascular architecture were further analysed by quantifying the morphometric differences in parameters describing the vascular network. Figure 3 A–E illustrates the differences in the frequency distributions of the parameters that have been measured. They are plotted as cumulative frequencies, which enables the form of the distribution, the median value, the minimum and maximum values, and differences between groups to be seen directly.

Figure 3A shows the distributions of the intervessel distances for the four tumours and the two normal tissues. It is plotted on a logarithmic scale because the data most closely approximate to a log normal distribution. There are very clear differences in the curves, both between the tumours and relative to the normal tissues. The HEC-1B xenograft is closer to the normal tissues than the syngeneic murine tumours. The vessels are often closer together in muscle than in gut serosa. Maximum spacing in gut subserosa is much shorter than in the tumours. The tumours have a much broader spectrum, higher median distances, and considerably larger intervessel distances. The median values and the numbers of measurements are listed in Table 2.

Figure 3B shows the quantitation of the distances between successive branches. Again, the data fit a log normal distribution and the six data sets are clearly different. On average, the normal tissue network shows a branching every 40–70 microns. There are clear differences in the range and the median values and the patterns of the distributions for the four tumours. Among the four tumours the branching is much less frequent in CaNT and SaS lesions and much more heterogeneous. In contrast, HEC-1B xenografts and CaX tumours are much closer to the normal tissues.

Interestingly, if we compute the number of tumour cells in a cylinder around each vessel in an intervessel segment, using half of the median values for intervessel distance and interbranch distance, and assuming an average cell diameter of 10 μm and a packing fraction of 70%, we obtain values of 260, 480, 2240 and 3700 cells per vessel in HEC-1B, CaX, SaS and CaNT tumours, respectively, compared to 170 cells per vessel in the subserosa of the gut. These data underline the massive differences in the number of cells being supplied with nutrients by individual microvessels among different types of tumours and the difficulties of flooding these cells with conventional cytostatic therapies as well as the potential avalanche effect of targeting the vessels rather than the tumour cells themselves.

Figure 3C shows the vessel diameters, again on a logarithmic scale. Again, six distinct patterns emerge with each tumour and normal tissue having a characteristic distribution of values. The two normal tissues differ by less than a factor of 1.3. The HEC-1B xenografts show wider vessels than the two normal tissues, but nevertheless significantly narrower ones than those from the rodent tumours.

Figure 4 illustrates the heterogeneity of vessel diameters within individual vessel segments, and the very large variability in the rodent tumours. Some values (not shown) are even higher than a 150% deviation from the mean, even within a relatively short vessel segment (less than 0.5 mm). The variability in the normal tissues is much smaller than in the tumours. These data are also shown as a cumulative frequency distribution in Figure 3D to illustrate the quantitative differences.

Figure 3E shows the last of the parameters that was measured, the branching angles of the vessels. These range from almost zero to almost 180° with a median value of about 70° in all the tumours and the normal tissues. This is the only parameter we have analysed that shows no significant differences between the group of murine tumours. The HEC-1B xenografts have a branching pattern least like that of the normal tissues. It is somewhat surprising that the distribution of the branching angles is nearly the same in both normal tissues even though the muscle vessels are mainly orientated longitudinally parallel to the muscle fibres.

Table 3 summarizes the significance levels. In general, the HEC-1B xenografts are closer to the normal tissue than to the other tumours, but significantly different from both, except for the branching angles.

### DISCUSSION

The significance of angiogenesis and the vascular architecture have been stressed in numerous studies of the structure and biological properties of the tumour vasculature and blood flow (Folkman, 1976; Jain, 1988; Folkman and D’Amore, 1966). The importance of the intervessel distances and the abnormal reactivity of the tumour microcirculation form the basis for therapeutic manipulations based on hypoxia or on proliferation gradients. This has been the driving force in the fields of radiobiology, radiosensitizer development and bioreductive drug development. It also underpasses the antiangiogenic and vascular targeting concepts, and the use of hyperthermia.

Despite the importance of angiogenesis in tumour biology, a puzzling divergence of opinion can be seen when reviewing the literature for the question of tumour specificity of the vascular *structure*. Since the early studies of Virchow (1863) and Thiersch (1865) there has been no doubt that, in general, tumour vessels are structurally different from vessels in normal tissues. Features like immature vessel walls with lack of muscle cells, missing adrenergic innervation and lymphatic drainage, discontinuous endothelial lining, as well as sinusoidal vessel plexuses are quoted in all papers. However, there is little evidence that the expression of these features is tumour type-specific (Falk, 1982). In a previous study (Konerding et al., 1992) we examined semiquantitatively, by means of transmission electron microscopy, the vessels of different human tumours (one undifferentiated melanoma, two leiomyosarcomas, one soft tissue sarcoma, two spindle cell sarcomas, one neurofibrosarcoma, one squamous cell carcinoma) xenografted into nude mice. We studied continuity of the endothelium, gaps, fenestrations, endothelial cell organelle contents, differentiation of the contact structure, basal lamina and pericytes. These fine features of the vascular network did not differ significantly between the individual tumour entities or between tumour types.

Characteristic vascular *architectures* for each tumour type were seen, however, many years ago by simple light microscopy (Lewis, 1927) in a variety of tumours: Margulis et al (1961) and Milne et al (1967) showed that tumours with comparable histology develop similar vascular patterns. In contrast, Solesvik et al (1982) demonstrated a different vascular pattern in five melanomas with the same general histological type. These divergent results might be due to differences between individual tumours arising in different patients or animals or, at least in part, due to the methods used. Nearly all studies of the tumour vascular architecture have been based on light microscopic and/or angiographic 2D methods, which enable a morphometric assessment of the gross network but cannot adequately describe the 3D microvascular architecture. This can best be done with 3D reconstructions of corrosion casts

 © Cancer Research Campaign 1999

(Malkusch et al., 1995). Reproducibility tests and tests for the possible errors indicate that a maximum result deviation of 2.5% may be expected (Malkusch et al., 1995).

In the present study, intervessel distances were measured as a parameter reflecting the vascular densities, which is comparable to the intercapillary distances measured by Less et al (1991). In addition, interbranch distances and branching angles were quantified as parameters determining the network organization and hierarchy. Furthermore, the vascular diameters and the variations of vascular diameter along a short segment were assessed because of their impact on blood flow and resistance. These provide a quantitative measure of the regularity or chaotic nature of the vascular bed and allow us to compare the different types.

The tumours we examined showed significant differences in the peripheral vasculature encircling each tumour, which was evident even at low power magnifications. This peri-tumoural vascular plexus is not specific to the subcutaneous tumour model we have used since it was described already in chamber implants of murine melanomas by Algire (1943). Goodall et al (1965) termed this the venous capsule, but it has also been described as the tumour vascular envelope (Grunt et al., 1986).

Imaging of the deeper microvascular networks of the different tumour lines revealed differences, which were obviously related to differences in the branching pattern, vascular density and vessel uniformity along its length. Quantitative analyses of the intervessel and interbranch distances proved that these differences are significant and confirmed that there is a characteristic vascular architecture in different types of tumour. The branching angles, however, did not differ significantly in the four examined tumour lines. This parameter is not a sensitive one, since the two normal tissues with completely different vascular patterns (skeletal muscle and the gut serosa) also have nearly identical distributions of the branching angles. The similarities in the branching angles might indicate a common basic architectural principle which cannot be transgressed, and which is retained across different types of normal tissues and tumours.

When comparing frequency distribution curves of the interbranch and intervessel distances of the tumour vasculature to those of the selected normal tissues, we see that the differences between the individual tumour types are as high or even higher than those between the two normal tissues studied, and also between tumours and normal tissues. It must be pointed out that these differences did not appear to be related to differences in size and rate of growth of the tumour.

The diameters of tumour vessels are wider than those in normal tissues. This is probably accompanied by high interstitial pressure because of the scarcity or complete absence of newly formed lymphatics. This may result in up to eightfold higher microvascular permeability of many tumour vessels (Gerlowski and Jain, 1986). This, in turn, has been associated with abnormal structural characteristics such as poor vessel wall differentiation, gaps and endothelial discontinuities.

Also in this case, the described differences in vascular diameters of the different tumour lines are not related to differences in size and rate of growth of the lesion: CaX tumours with a more than fourfold bigger size had smaller vessel diameters than CaNT tumours. Moreover, fast-growing tumours of comparable sizes (CaNT and HEC-1B tumours) showed significant differences in vascular diameters.

Vascular diameters varied considerably in the three murine tumours. In the human HEC-1B xenografts the diameters were less variable. This could be due to differences in the origin of the tumour line (human vs rodent) or to the difference in host mice (nude vs CBA). This is under investigation. We have recently demonstrated that the variations in vessel diameter along a segment can be grossly altered by the overexpression of a single angiogenic growth factor. Indeed, we have observed a massive increase in the variability in vessel diameter in xenografts originated by clones of HEC-1B cells transfected with the human FGF2 cDNA and that overexpress and secrete significant amounts of the growth factor (Konerding et al, 1998).

These data demonstrate the interdependence of the tumour cells and the vessel walls components to create a characteristic vasculature in our experimental model. Our findings suggest that the vascular architecture of an experimental tumour does not depend on the size and/or the rate of growth of the lesion, but rather on features characteristic of the tumour cell type. These observations have profound implications in the use of experimental tumour models for the evaluation of novel anti-neoplastic therapies. Indeed, vascular heterogeneity may have a marked influence on the microenvironmental gradients of the different tumour types and, consequently, on their response to all forms of experimental therapy. Large intervessel distances and poor blood flow lead to hypoxic resistance to radiotherapy and to chemoresistance. The latter is further worsened by the high interstitial pressure, diminishing the diffusion rates of large molecules through the interstitial space. Similar implications might be hypothesized also in the therapy of human tumours in the case that such a tumour type-dependent heterogeneity of the vascular architecture will be demonstrated in biopsies of human neoplasms.

This study emphasizes the need for a better understanding of the architecture of the microvasculature in solid tumours. The information obtained in this way must then be correlated with knowledge of the microenvironmental factors that influence tumour cell viability growth characteristics and susceptibility to various anticancer approaches.

### ACKNOWLEDGEMENTS

This study was supported by grants of the European Community (Human Capital and Mobility Program ERB CHRX-CT9-0593: Mechanisms for the Regulation of Angiogenesis) to MK, from the Robert-Müller-Stiftung für Herz-Kreislaufforschung to MK, from the Cancer Research Campaign UK, and from Associazione Italiana per la Ricerca sul Cancro to MP.

### REFERENCES

Algire GH (1943) Microscopic studies of the early growth of a transplantable melanoma of the mouse using the transparent chamber technique. *J Natl Cancer Inst* **4**: 13–22

Bicknell R (1994) Vascular targeting and the inhibition of angiogenesis. *Ann Oncol* **5**: 45–50

Davidson SE, Ngan R, Wilks DP, Moore JV and West CM (1994) A comparison of four methods for assessing tumor vascularity in carcinoma of the cervix. *Int J Oncol* **5**: 639–645

Denekamp J (1984) Vascular endothelium as the vulnerable element in tumours. *Acta Radiol Oncol* **23**: 217–225

Endrich B and Vaupel P (1998) The role of the microcirculation in the treatment of malignant tumors: facts and fiction. Morphological aspects of tumor angiogenesis and microcirculation. In *Blood Perfusion and Microenvironment of Human Tumors, Implications for Clinical Radiooncology*, Molls M and Vaupel P, (eds), pp. 19–40. Springer: Berlin

© Cancer Research Campaign 1999

Falk P (1982) Differences in vascular pattern between the spontaneous and the transplanted C3H mouse mammary carcinoma. *Eur J Cancer Clin Oncol* **18**: 155–165

Folkman J (1976) The vascularization of tumors. *Sci Am* **234**: 58–64, 70–73

Folkman J and D'Amore PA (1996) Blood vessel formation: what is its molecular basis? *Cell* **87**: 1153–1155

Gerlowski LE and Jain RK (1986) Microvascular permeability of normal and neoplastic tissues. *Microvasc Res* **31**: 288–305

Goodall CM, Sanders AG and Shubik P (1965) Studies of vascular patterns in living tumors with a transparent chamber inserted in the hamster cheek pouch. *J Natl Cancer Inst* **35**: 497–521

Grunt TW, Lametschwandtner A, Karrer K and Staindl O (1986) The angioarchitecture of the Lewis lung carcinoma in laboratory mice. A light microscopic and scanning electron microscopic study. *Scan Electron Microsc* 557–573

Jain RK (1988) Determinants of tumor blood flow: a review. *Cancer Res* **48**: 2641–2658

Konerding MA, Fait E, Dimitropoulou C, Malkusch W, Ferri C, Giavazzi R, Coltrini D and Presta M (1998) Impact of Fibroblast Growth Factor-2 on tumor microvascular architecture: a tridimensional morphometric study. *Am J Pathol* **152**: 1607–1616

Konerding MA, Steinberg F and Budach V (1989) The vascular system of xenotransplanted tumors: scanning electron and light microscopic studies. *Scanning Microsc* **3**: 327–336

Konerding MA, Steinberg F, van Ackern C, Budach V and Streffer C (1992) Comparative ultratructural studies of the vascularity in different human xenografted tumours. In: *Immunodeficient Mice in Oncology. Contributions to Oncology*, Fiebig HH and Berger DP (eds), pp. 169–179. Karger: Basel

Less JR, Skalak TC, Sevick EM and Jain RK (1991) Microvascular architecture in a mammary carcinoma: branching patterns and vessel dimensions. *Cancer Res* **51**: 265–273

Lewis WH (1927) The vascular patterns of tumors. *Johns Hopkins Hosp Bull* **41**: 156–175

Malkusch W, Konerding MA, Klapthor B and Bruch J (1995) A simple and accurate method for 3-D measurements in microcorrosion casts illustrated with tumour vascularization. *Anal Cell Pathol* **9**: 69–81

Margulis AR, Carlsson E and McAllister WH (1961) Angiography of malignant tumors in mice. *Acta Radiol* **56**: 179–192

Milne EN, Margulis AR, Noonan CD and Stoughton JT (1967) Histologic type-specific vascular patterns in rat tumors. *Cancer* **20**: 1635–1646

Solesvik OV, Rofstad EK and Brustad T (1982) Vascular structure of five human malignant melanomas grown in athymic nude mice. *Br J Cancer* **46**: 557–567

Thiersch C (1865) *Der Epithelialkrebs namentlich der Haut*. Engelmann: Leipzig

Thomlinson RH and Gray LH (1955) The histological structure of some human lung cancers and the possible implications for radiotherapy. *Br J Cancer* **9**: 539

Vaupel P and Gabbert H (1986) Evidence for and against a tumor type-specific vascularity. *Strahlenther Onkol* **162**: 633–638

Virchow R (1863) *Die krankhaften Geschwülste*. Berlin: Hirschwald

Warren BA (1979) Tumor angiogenesis. In: *Tumor Blood Circulation. Angiogenesis, Vascular Morphology and Blood Flow of Experimental and Human Tumors*, Peterson HI (ed), pp. 47–75. CRC Press: Boca Raton

 *© Cancer Research Campaign 1999*
